# Contralateral anterior cruciate ligament injury after anterior cruciate ligament reconstruction: a case controlled study

**DOI:** 10.1186/1758-2555-4-46

**Published:** 2012-12-10

**Authors:** Junsuke Nakase, Hiroyuki Tsuchiya, Katsuhiko Kitaoka

**Affiliations:** 1 Department of Orthopaedic Surgery, School of Medicine, Kanazawa University, 13-1 Takara-machi, Kanazawa 920-8641, Japan; 2 Department of Orthopaedic Surgery, Kijima Hospital, 41-1 Matsutera-machi, Kanazawa, 920-0011, Japan

**Keywords:** ACL reconstruction, Risk factors, Contralateral ACL injury

## Abstract

**Purpose:**

The purpose of this present study was to examine contralateral ACL injury cases after ACL reconstruction, to determine the characteristics of such injuries.

**Methods:**

We performed a retrospective analysis of 24 patients with contralateral ACL injury after ACL reconstruction. The control group consisted of 200 cases with unilateral ACL injury. The following were examined in the contralateral group: timing of the contralateral ACL injury, and the situations of the initial and contralateral ACL injuries. The following items were compared between the contralateral and control groups: age at the time of initial injury, level of competitive sports using Tegner activity scores, knee anterior laxity (KT-1000), and the ratio (%) of affected to unaffected legs in the strengths of the knee extensor and flexor muscles 6 months after surgery.

**Results:**

Examination of injury situations showed that approximately 70% of the contralateral group was injured in situations similar to those at their initial injuries. There were no significant differences between the two groups in age at the time of initial injury , Tegner activity scores, knee anterior laxity, and the strengths of the knee extensor, flexor muscles and H/Q ratio 6 months after reconstruction. But, the age at the time of initial injury trended to be low in contralateral group.

**Conclusions:**

Knee anterior laxity and muscle weakness of the reconstructed legs six months following surgery were not individually related to contralateral ACL injury occurring approximately two years after surgery.

## Introduction

In recent years, there has been an increase in the incidence of anterior cruciate ligament (ACL) injury accompanying the increase in people playing sports. The incidence of primary ACL injury has been reported as 1.5% to 1.7% per year in a healthy athletic population [1,2]. The majority of ACL injuries occur during landing, deceleration, or side cutting maneuvers [3]. The athlete’s desire to return to sport is cited as a major indication for ACL reconstruction surgery [4]. Previous studies reported that the rate of return to sports was 70-80% [5,6]. However, there are some complications after ACL reconstruction. Contralateral ACL injury is one of the most serious complications after reconstruction [7,8].

The etiology of ACL injuries is complex. Although different factors have been implicated in the risk of ACL injury, it is still not thoroughly understood why some individuals are at higher risk than others. These risk factors can be defined as intrinsic (e.g. gender [9], size of intercondylar notch [10]) and extrinsic (e.g. proprioception [11], quadriceps-to-hamstring ratio [12]). There is no consensus in the literature regarding the relevance of different risk factors for ACL injury [13]. There is also insufficient knowledge about risk factors for contralateral ACL injury. The objectives of this present study were to examine contralateral ACL injury cases after ACL reconstruction, to determine the characteristics of such injuries. Our hypothesis is that the contralateral ACL injury cause by muscle weakness and/or instability of the reconstructed knee at 6 month after ACL reconstruction.

## Materials and methods

The study was reviewed and approved by the ethics committee of our university.

The subjects were 24 patients (5 males and 19 females) who underwent ACL reconstruction at our institution between October 1998 and March 2006. They had returned to playing sports after reconstruction and had an injured contralateral ACL. The control group consisted of 200 cases (45 males and 155 females) with unilateral ACL injuries. ACL reconstruction was performed by the method of Howell [14]. Single folded semitendinosus tendon and gracilis tendon was used and single route non-anatomic reconstruction was performed. Tendon graft fixation was achieved by cross pins on the femoral side and by a washer plate and screw on the tibial side. Postoperatively, bracing was not used. The patients were permitted to perform weight-bearing ambulation from the day after surgery, and range of motion training was started. The patients were permitted to return to playing sports if they met the following conditions: (1) 6 months or more had passed since the surgery, (2) there was neither swelling of the knee joint nor limited range of motion, (3) there was no instability by objective and subjective assessment, and (4) for the strengths of the knee extensor and flexor muscles, the ratio of affected to unaffected legs was over 80%.

The following were examined in the contralateral group at the initial injury and at the re-injury: injury situation (e.g., landing a jump and cutting motion) and duration of reconstruction to contralateral injury. The following items were compared between the control group and contralateral group: age at the time of initial injury, level of competitive sports using Tegner activity scores, knee anterior laxity using KT-1000 when patients returned to sports activity and the ratio (%) of affected to unaffected legs in the strengths of the knee extensor and flexor muscles 6 months after reconstruction. Knee laxity was measured by the side-to-side differences in displacement on manual maximum testing. Muscle strengths were measured with a Biodex dynamometer and the values were compared using an angular velocity of 180 degrees/sec. The Logistic regression analysis was used to determine statistical differences. Results were considered significant at the 95% confidence interval level for all statistical analyses. Statistical analysis was performed using SPSS for Windows software v 11.0 (SPSS Inc, Chicago, IL).

We included 24 patients with bilateral ACL injury and 200 patients with unilateral ACL injury in 575 patients that performed ACL reconstruction from October 1998 to March 2006. About knee ext. muscle strength (%). The average is 100 and standard variation is 20, α error is 0.05, β error is 0.20( power=0.8), sample size is 200, 20 for detected the difference of two group 10% of mean.

## Results

In the contralateral group 92% had non-contact injuries at the initial injury and 88% had non-contact injuries at contralateral injury. In the control group 85% had non-contact injuries. The examination of injury situation showed that approximately 70% of the contralateral group was injured in situations similar to those at their initial injuries. Average time from reconstruction to contralateral ACL injury was 22.5 months. There were no significant differences in the age at the time of injury, Tegner activity score, knee anterior laxity and knee extensor and flexor muscle strength. But, the age at the injury trended to be low in contralateral group in comparison with control group (Table 1). Therefore, there being two differences only in 5% for knee ext. muscle strength(%) consider power more than 0.8. And there is no difference contralateral group and control group. Similarly, there were no differences for knee flex. muscle strength and knee laxity.

**Table 1:** Comparison between contralateral group and control group OR: odds ratio

	**contralateral group**	**control group**	**P value**	**OR**
Age at the time of initial injury	17.5±4.0	19.3±4.4	0.098	1.127
Tegner activity score	7.2±0.8	7.0±0.7	0.192	0.655
Knee laxity (mm)	1.2±1.6	1.0±1.2	0.396	0.842
Knee ext. muscle strength (%)	104.9±19.2	99.9±16.9	0.801	0.988
Knee flex muscle strength (%)	99.6±20.1	98.3±21.1	0.908	0.994
H/Q ratio	0.96±0.20	0.98±0.25	0.923	1.626

## Discussion

Souryal [1] reported the occurrence of contralateral ACL rupture after ACL reconstruction in 45 patients (4.1%) of 1120 patients who underwent ACL reconstruction. Salmon [7] examined 675 patients who underwent ACL reconstruction. They conducted a phone survey 5 years after reconstruction surgery. There were 35 patients (5.7%) who developed a contralateral ACL rupture. Wright [15] conducted a survey for 2 years after ACL reconstruction and reported that 3% of their subjects had a contralateral ACL injury. Contralateral ACL injury is one of the most serious complications after ACL reconstruction for patients and surgeons [16]. Recently, Wright [8] reported systematic review for contaralateral ACL rupture at five years or more following ACL reconstruction. The systematic review demonstrates that the risk of ACL tear in the contralateral knee was 11.8%.

Previous studies have evaluated bilateral ACL injuries to try to determine their risk factors [7,17,18]. Souryal [1] reported that the risk factor of contralateral ACL injury was young age at the time of initial injury. Furthermore, Pinczewski [18] showed that individuals who are young at the time of ACL reconstruction have a greater risk of a contralateral ACL injury during follow up. In our study, the age at the time of initial injury in contralateral group trended to be low compare with control group. Patients with a ruptured ACL are likely to have a higher risk of having various intrinsic factors that make patients more susceptible to an ACL injury. Young patients are probably more likely to return to sports activity than older individuals with first-time ACL injury.

Salmon [7] analyzed the type of primary ACL injury (contact or non-contact injury), activity level according to the International Knee Documentation Committee (IKDC) scale, gender, graft type, family history of ACL injury, articular surface damage, presence of meniscal injury, history of meniscectomy, and correlation to contralateral ACL injury. The only significant predictor for a contralateral ACL injury was a return to sports activity of level 1 or 2. In our study, most of the cases had undergone reconstruction to return to playing competitive sports. Only a small number professional athletes were in our study, which could have resulted in an insignificant difference in Tegner activity score.

For graft choice, a significant difference has been demonstrated between patients who have undergone ACL reconstruction with hamstring tendon autograft compared to patients who have undergone ACL reconstruction with patellar tendon, in terms of contralateral ACL injury rate. There were significantly more contralateral ACL injuries in the patellar tendon group at 10-year follow up [18].

There has not been any report on the examination of knee laxity and muscle strength of reconstructed legs in patients with contralateral ACL injuries after ACL reconstruction. At first, our study was begun with the hypothesis that the risk factors for contralateral ACL injury are knee laxity and insufficient muscle strength of the reconstructed knee. However, our results indicated that when the patients returned to playing sports, there was no significant difference between the contralateral group and the control group in the knee laxity and strength of the knee extensor and flexor muscles of the reconstructed legs.

We also focused on the injury situation. We found that most of the cases of contralateral ACL injuries were non-contact injuries at the time of initial and re-injury. The re-injuries of approximately 70% of the cases occurred under similar situations as their initial injuries. These results may suggest that the patients with initial non-contact ACL injuries tended to develop non-contact injuries again if neuromuscular control of the legs was not improved. One of the risk factors of contralateral ACL injury is considered to be a non-contact injury at the initial injury. To prevent non-contact injuries, the following are thought to be effective: physiological and kinesiological approaches suitable to each individual and prevention programs for ACL injuries such as neuromuscular training [19-21].

To our knowledge, there has not been any report on risk factors for contralateral ACL injury focusing on knee laxity and muscle strength of the reconstructed legs. Our study suggest that knee laxity and muscle weakness of the reconstructed legs 6 months following surgery were not individually related to contralateral ACL injury occurring approximately 2 years after surgery.

## Conclusion

The age at the time of initial injury trended to be low in contralateral group. However, the sports activity level did not differ significantly between these groups. In addition, knee laxity and muscle weakness of the reconstructed legs 6 months following surgery were not individually related to contralateral ACL injury occurring approximately 2 years after surgery.

## Competing interests

The authors declare that they have no competing interests.

## Authors’ contributions

JN is the principal investigator. KK treated all the patients. All authors co-wrote the paper and discussed the results for the manuscript preparation. All authors have read and approved the final manuscript.
